# Long term safety of targeted internalization of cell penetrating peptide crotamine into renal proximal tubular epithelial cells *in vivo*

**DOI:** 10.1038/s41598-019-39842-7

**Published:** 2019-03-01

**Authors:** Joana Darc Campeiro, Wendy Dam, Gabriela Guilherme Monte, Lucas Carvalho Porta, Lilian Caroline Gonçalves de Oliveira, Marcela Bego Nering, Gustavo Monteiro Viana, Fernando Cintra Carapeto, Eduardo Brandt Oliveira, Jacob van den Born, Mirian A. F. Hayashi

**Affiliations:** 1 Departamento de Farmacologia, Escola Paulista de Medicina (EPM), Universidade Federal de São Paulo (UNIFESP), São Paulo, SP Brazil; 2 Department Nephrology, University Medical Center Groningen, University of Groningen, Groningen, The Netherlands; 3 Departamento de Biofísica, Escola Paulista de Medicina (EPM), Universidade Federal de São Paulo (UNIFESP), São Paulo, SP Brazil; 4 Departamento de Bioquímica, Escola Paulista de Medicina (EPM), Universidade Federal de São Paulo (UNIFESP), São Paulo, SP Brazil; 5 Departamento de Patologia, Escola Paulista de Medicina (EPM), Universidade Federal de São Paulo (UNIFESP), São Paulo, SP Brazil; 6 Departamento de Bioquímica e Imunologia, Universidade de São Paulo (USP-FMRP), Ribeirão Preto, Brazil

## Abstract

Activated proximal tubular epithelial cells (PTECs) play a crucial role in progressive tubulo-interstitial fibrosis in native and transplanted kidneys. Targeting PTECs by non-viral delivery vectors might be useful to influence the expression of important genes and/or proteins in order to slow down renal function loss. However, no clinical therapies that specifically target PTECs are available at present. We earlier showed that a cationic cell penetrating peptide isolated from South American rattlesnake venom, named crotamine, recognizes cell surface heparan sulfate proteoglycans and accumulates in cells. In healthy mice, crotamine accumulates mainly in kidneys after intraperitoneal (*ip*) injection. Herein we demonstrate for the first time, the overall safety of acute or long-term treatment with daily *ip* administrated crotamine for kidneys functions. Accumulation of *ip* injected crotamine in the kidney brush border zone of PTECs, and its presence inside these cells were observed. In addition, significant lower *in vitro* crotamine binding, uptake and reporter gene transport and expression could be observed in syndecan-1 deficient HK-2 PTECs compared to wild-type cells, indicating that the absence of syndecan-1 impairs crotamine uptake into PTECs. Taken together, our present data show the safety of *in vivo* long-term treatment with crotamine, and its preferential uptake into PTECs, which are especially rich in HSPGs such as syndecan-1. In addition to the demonstrated *in vitro* gene delivery mediated by crotamine in HK-2 cells, the potential applicability of crotamine as prototypic non-viral (gene) delivery nanocarrier to modulate PTEC gene and/or protein expression was confirmed.

## Introduction

Loss of renal function is often related to interstitial fibrosis and tubular atrophy^1^. Many attempts to slow down or even reverse the interstitial fibrosis are aimed at the level of (myo)fibroblasts or at the level of matrix remodeling^2–5^. Recently, major evidence suggests that tubulo-interstitial fibrosis is the consequence of chronic activation of tubular cells, mainly of proximal tubular epithelial cells (PTECs)^6^. This tubular activation is secondary to ischemia, salt- and acid-loading, proteinuria or exposure to toxic drugs, or is due to immunological signals during renal inflammation, injury or transplantation^7–10^. Related to these activating noxi, changes of PTECs proteome expression profile are reported, among which are the cell membrane receptors, cytoskeletal elements and signaling pathways, and production of a wide array of soluble mediators, ranging from growth factors and chemokines to complement factors and reactive oxygen species^11^. In a vicious circle, recruited myeloid cells strengthen chronic PTEC activation and contribute to interstitial fibrosis^12^. Although a direct contribution of epithelial to mesenchymal transition to renal fibrosis seems not very likely, epithelial involvement in renal fibrosis via instruction of recruited interstitial myeloid and mesenchymal cells has been convincingly shown in renal transplantation ischemia-reperfusion, proteinuria and renal obstruction^13,14^.

Cornerstone for current treatment of renal function loss is based on lowering the blood pressure and proteinuria, mainly by targeting the renin-angiotensin-aldosterone system^15^. Although this approach proved to effectively slow down end-stage renal disease, there is still no cure for renal fibrosis, most probably because the current treatments are not aimed at tubular, but rather at vascular and glomerular levels. At present, no clinical therapies that specifically target the PTECs are available.

In this report, we evaluate the use of the cell penetrating peptide (CPP) crotamine as a PTEC specific non-viral delivery nanocarrier. CPPs are molecules that display the ability to enter and efficiently carry into eukaryotic cells, a number of biologically active and therapeutically relevant molecules, including DNA and potentially chemical drugs as well^16,17^.

Crotamine is a positively-charged 42 amino acid residues polypeptide, isolated from the South American rattlesnake *Crotalus durissus terrificus* venom, with CPP properties, as the characteristic ability of crossing the lipid bilayer of cellular membranes and of transporting cargo into cells^18–20^. In addition, crotamine is non-toxic to cells at low micromolar concentrations, and thereby, it can be safely used to transfect mammalian cells *in vitro* and *in vivo*^19–21^. The mechanism by which crotamine or crotamine-DNA complexes enter tumor cells involves the binding to cell surface heparan sulfate proteoglycans (HSPGs), which is followed by endocytosis^18^. The ability of crotamine to permeabilize endosomal/lysosomal vesicles confers an additional and unique advantage for this polypeptide, as gene nanocarrier^18–20,22^. Furthermore, crotamine contains a nuclear retention motif, which after liberation from endosomal/lysosomal vesicles, guides crotamine to the nucleus of transfected cells^23,24^. Combined, these characteristics make crotamine a unique and ideal candidate as nanocarrier for non-viral delivery of (therapeutic) molecules into PTECs, which are especially rich in HSPGs such as syndecan-1 (Synd-1)^25^.

Proteoglycans are glycoconjugates of glycosaminoglycan chains covalently attached to a protein core^26^. Syndecans comprise a major family of cell surface HSPGs. The mammalian syndecan family consists of 4 distinct members *i.e*. syndecan 1–4, all encoded by distinct genes. Almost all cell types express one or more syndecans and their expression is spatio-temporally regulated.

Synd-1 is primarily expressed on epithelial cells such as PTECs, but it is also present in hepatocytes and plasma cells. In general, Synd-1 regulates the biological activity of ligands by affecting their stability, conformation, oligomerization, compartmentalization and cellular uptake, and thereby, Synd-1 modulates the concentration, distribution and activity of its ligands. Synd-1 mostly acts as a co-receptor by increasing the responsiveness to external stimuli^27^, and as an autonomous endocytosis receptor^28,29^. We earlier published on the significance of renal tubular Synd-1 in tubular regeneration^30^, and as a docking station for complement factors^31^.

In this report, we describe the *in vivo* specific internalization of crotamine administrated by intraperitoneal (*ip*) route into PTEC, which are especially rich in HSPGs such as Synd-1. The applicability and safety of long-term *ip* administration of crotamine in mice was indicated by the absence of any significant adverse effects, as assessed by histopathological analysis and evaluation of blood and urine biochemical markers of kidney function of mice receiving crotamine for three weeks. In addition, the importance of Synd-1 for crotamine and crotamine-DNA complex internalization into PTECs was verified *in vitro* using the wild-type and Synd-1 deficient PTECs. Taken together, these findings open possibilities of using crotamine as a non-viral nanocarrier vector in order to specifically deliver therapeutic DNA and/or drugs into PTECs *in vivo*.

## Results

### Safety of long-term *in vivo* treatment with crotamine and its clearance by the kidneys

Continuous daily treatment with crotamine (1 μg/animal) by *ip* injection showed no significant change in average body weight of crotamine-treated compared to control mice receiving vehicle, at the end of 21 days treatment, which was also accompanied by non-obvious general influence in animal behavior, condition or healthy state. The similar weight of the organs and bone (femur) size, between the crotamine-treated and control group animals receiving saline, also confirmed the overall safety of this treatment (Table 1).

**Table 1:** Main organs and body weight, femur size, food and water intake and renal function parameters.

		Control	Crotamine
Kidney (g)		0.34 ± 0.01	0.33 ± 0.01
Liver (g)		1.14 ± 0.07	1.11 ± 0.09
Heart (g)		0.14 ± 0.01	0.13 ± 0.01
Femur (cm)		1.58 ± 0.01	1.56 ± 0.02
Body Weight (g)		25.78 ± 0.71	24.88 ± 0.72
Food Intake (g/24 h/5 mice)		83.50 ± 2.08	76.32 ± 1.95*
Water Intake (mL/24 h/5 mice)		21.62 ± 0.72	18.39 ± 0.93*
Creatinine (mg/dL)	Plasma	2.84 ± 0.61	2.39 ± 0.42
	Urine	3.86 ± 0.07	2.91 ± 0.48
Uric acid (mg/dL)	Plasma	1.55 ± 1.01	1.89 ± 0.64
	Urine	2.30 ± 0.53	3.73 ± 0.40

Measurements of body and organs weights, femur size and plasma and urine biochemical biomarkers analyses were performed at the end of the treatment for 21 days with crotamine (1 μg/animal/day). For food and water intake assessment measures were performed at every 4 days, and the presented data correspond to the final mean value of consumption after the end of 21 days treatment period.

*p < 0.05 for t-Student statistical test. Results are expressed as mean ± SEM (N = 5 per group).

On the other hand, despite the relative smaller intake of food and water observed for the crotamine-treated compared to the control group, the levels of the biochemical biomarkers for renal function, namely creatinine and uric acid, were found unchanged in both plasma and urine of both crotamine-treated or negative control groups which received vehicle (Table 1).

**Figure 1:**
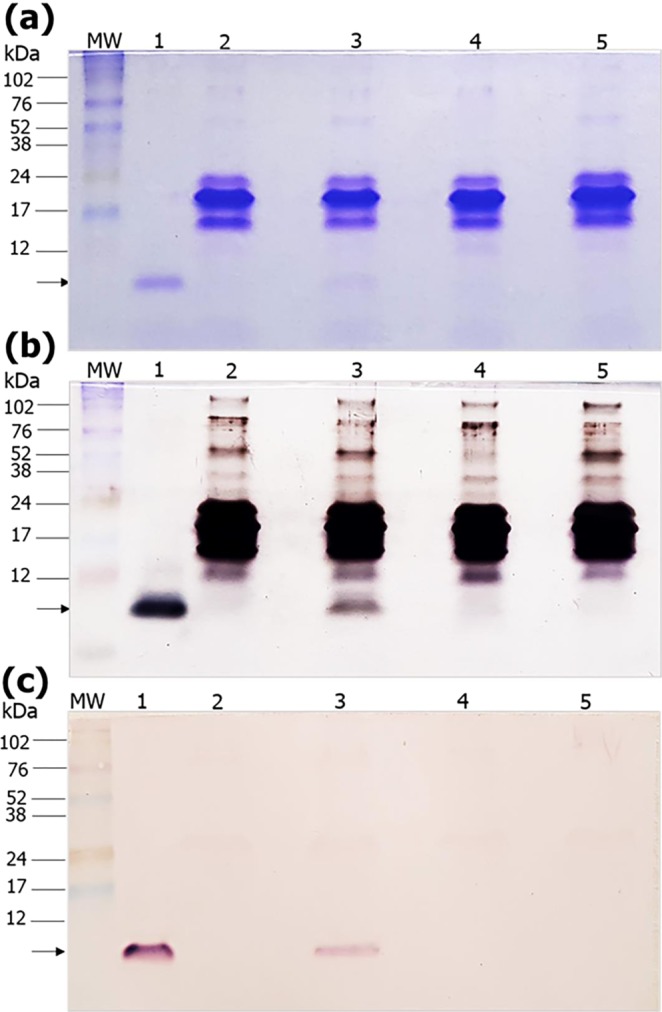
SDS-PAGE and Western blot analysis of mice urine samples. Urine of mice treated daily with vehicle saline or crotamine, by intraperitoneal (*ip*) route 1.5 h before urine collection, were applied to a 15% SDS-PAGE before the (**a**) staining with Coomassie blue, (**b**) silver staining, or (**c**) Western blot analysis with anti-crotamine antibody followed by development with alkaline phosphatase substrates NBT/BCIP. The presence of a protein band of about 5 kDa, corresponding to the full-length crotamine, is indicated by the arrows. MW: molecular weight markers (kDa), lane 1: native crotamine (100 ng), lane 2: urine of drug-naïve mouse receiving a single injection of vehicle saline (100 µL/animal), lane 3: urine of drug-naïve mouse receiving a single injection of native crotamine (30 µg/100 µL/animal), lane 4: urine of negative control mice treated daily with vehicle (100 µL/animal) for 21 days, and lane 5: urine of mice treated daily with crotamine (1 μg/100 µL/animal) for 21 days. In each lane, 5 µL of urine sample were loaded. The gels (**a** and **b**) and blot (**c**) are cropped along the edges. Complete uncropped blots/gels are presented in Supplementary Figure 3.

We earlier showed that *ip* injected crotamine accumulates into the kidneys of healthy mice^20,32^, as also demonstrated by others^33^. Herein, Coomassie blue or silver stained SDS-PAGE and Western blot analysis allowed confirming the presence of intact full-length crotamine in the urine of mice receiving crotamine (30 µg/animal) by *ip* route, 1.5 h before euthanasia (Fig. 1). The integrity of the full-length crotamine was also confirmed by mass spectrometry (MS) analysis (Supplementary Fig. S1) indicating glomerular filtration and urinary excretion of long-term *ip* injected native crotamine (Fig. 1) and/or of acute *ip* injected fluorescently-labeled Cy3-crotamine (Supplementary Fig. S2). The specificity of the anti-crotamine antibody employed in the present study was also confirmed by ELISA assay (Supplementary Fig. S3). In addition, the urinary protein excretion was analyzed by SDS-PAGE allowed observing that either long-term or acute crotamine administration exhibit no effect in proteinuria, as similar whole-urine protein profile was observed for mice receiving crotamine or vehicle (Fig. 1A,B; Supplementary Fig. S4), indicating that glomerular filtration and tubular reabsorption were not affected by the long-term treatment (for 21 days) with low doses (1 μg/animal/day) or high single dose (30 μg/animal) of acute *ip* administration of crotamine. However, the MS analyses did not allow detecting crotamine in the urine of mice treated daily with low doses of crotamine (1 μg/animal).

**Figure 2:**
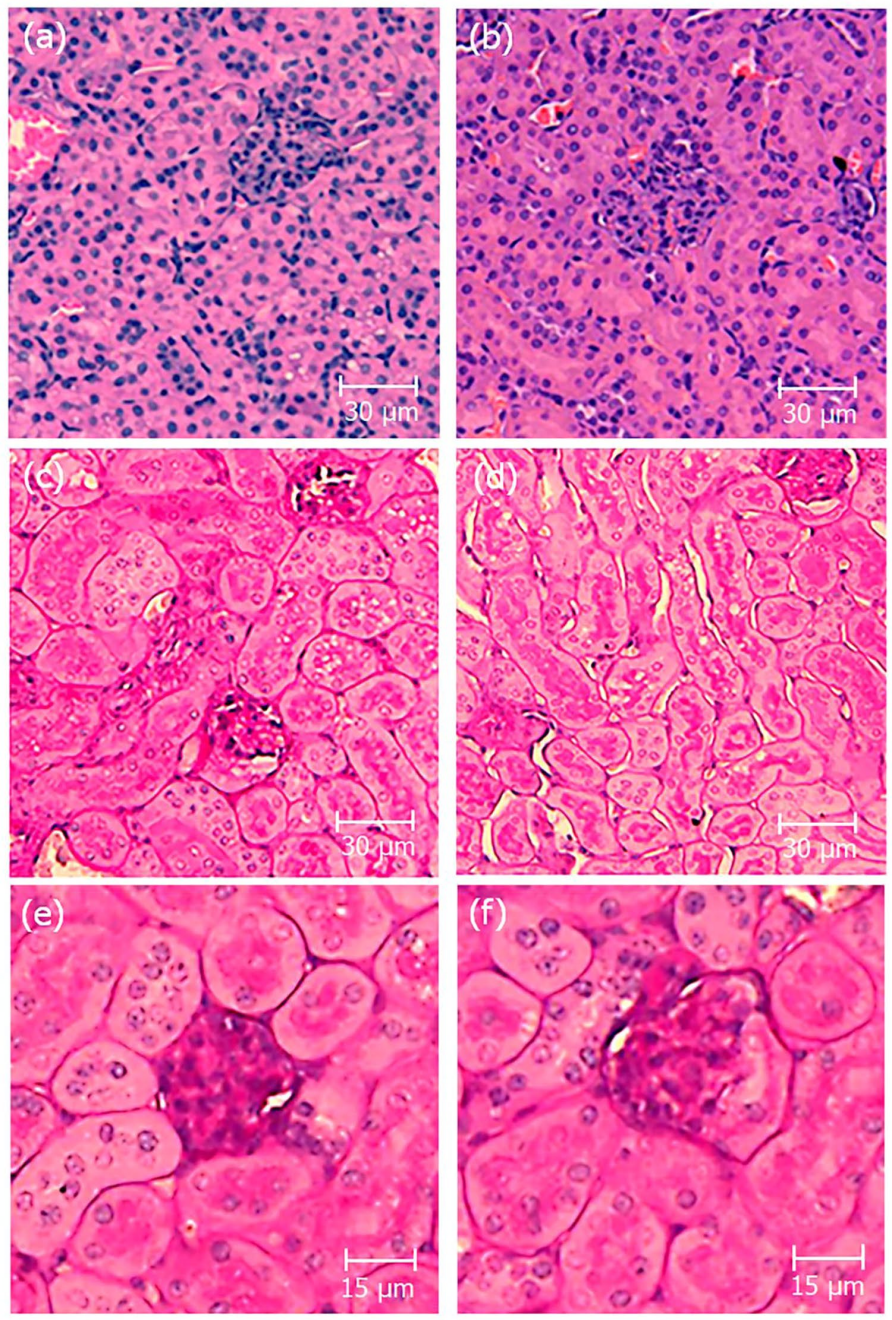
Histopathological analysis of kidney sections. Representative pictures of kidney sections stained by hematoxylin/eosin (H&E) (**a**,**b**) and periodic acid-Schiff (PAS) (**c**–**f**). Kidneys from control receiving vehicle (**a**, **c** and **e**) and crotamine-treated (**b**,**d** and **f**) mice. The glomerulus structure is shown in the magnified images (**e** and **f**). No evidence of histopathological lesions was noticed in the kidney sections analyzed by trained pathologists. Bar = 30 μm (**a**–**d**) and 15 μm (**e**,**f**).

Tissue morphology assessment of mice kidneys by histological analysis after classical hematoxylin/eosin (H&E) and periodic acid-Schiff (PAS) staining highlighted the absence of any significant observable alterations in kidney tissues sections of long-term crotamine-treated mice, as representatively shown in Fig. 2. It is also important to note the absence of any remarkable injuries or any other important alterations in other tissues as liver, lung, spleen and heart of mice treated daily with crotamine (1 μg/animal) by *ip* route, for 21 days, which was exactly the same condition previously adopted by us for the antitumoral therapy with this peptide^34,35^.

### Crotamine internalization in renal proximal epithelial cells *in vivo*

**Figure 3:**
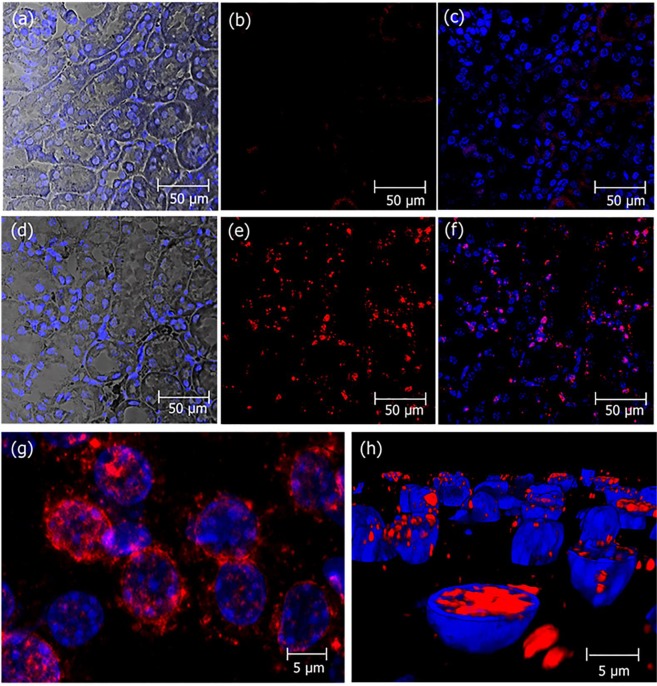
Localization of crotamine in kidneys PTECs of mice receiving daily crotamine by *ip* injections during 21 days. Kidney sections from mice receiving vehicle saline (**a–c**) or native crotamine (1 µg/animal), daily, for 21 days (**d–h**). The perinuclear localization of crotamine (red) is shown in the magnified picture (**g**). The presence of crotamine (red) inside the nuclei was confirmed by the three-dimensional reconstitution of cell nucleus as shown in (**h**). Differential interference contrast (DIC) with nuclei stained with DAPI (blue) (**a** and **d**), and crotamine immunorecognized by rabbit anti-crotamine antibody followed by signal amplification by the Tyramide Signal Amplification (TRITC-labeled tyramide solution, tetramethyl rhodamine system, in red) (**b,c,e,f,g** and **h**). Overlay of red and blue fluorescence (**c,f,g** and **h**). Bar = 50 μm (**a–f**) and 5 μm (**g, h**).

Immunohistochemical analysis of the same kidneys allowed visualizing native crotamine administered at low doses (1 µg/animal) by *ip* injections daily, during 21 days. Crotamine was localized into the PTECs, indicating the *in vivo* uptake of crotamine by these cells (Fig. 3). As a control for antibody specificity, immunohistochemical analysis of the same kidneys with rabbit anti-crotamine, blocked with native crotamine, for 1 h prior to staining procedure, showed a completely negative staining.

**Figure 4:**
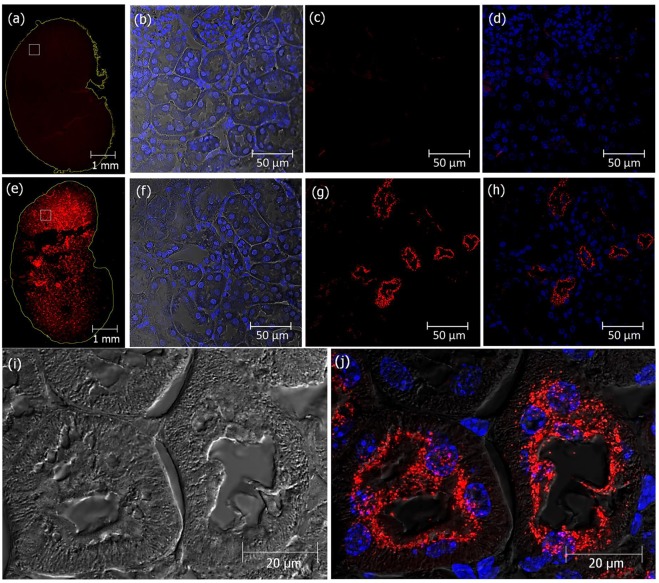
Cy3-labeled crotamine is reabsorbed from pro-urine by PTECs. Kidney sections from mice receiving vehicle (**a**–**d**) or a single *ip* administration of fluorescently-labeled Cy3-crotamine (5 µg/animal), 2 h before animal euthanasia (**e–j**). Fluorescence microscopy of transversal section of whole kidney (**a** and **e**), in which the boxes correspond to the areas magnified (as presented in **b**,**c**,**d**,**f**,**g** and **h**). Zoom out of kidney transversal section of mice showing the presence of Cy3-crotamine in the brush borders area of PTECs (**i**,**j**). Differential interference contrast (DIC) showing kidney tissue structure with nuclei stained with DAPI (blue) (**b,f**), and Cy3-crotamine stained in red (**a**,**c**,**d**,**e**,**g**,**h** and **j**). Differential interference contrast (DIC) image (**i**), and overlay of red and blue fluorescence with DIC (**d**,**h** and **j**). Bar = 1 mm (**a**,**e**), 50 μm (**b–d**, **f–h**), and 20 μm (**i, j**).

Fluorescent analysis of sections of mice kidney collected 2 h after acute single dose *ip* administration of fluorescently labeled Cy3-crotamine (5 µg/animal), employing In Cell Analyzer for whole tissue section analysis and also confocal microscopy for magnified pictures allowed observing the presence of crotamine localized in the microvilli-covered luminal surface (brush borders) of PTECs in several regions of the kidney (Fig. 4).

### *In vitro* assays for crotamine using cultured HK-2 PTEC cells

Since crotamine mainly enters cells via HSPGs^18^ and Synd-1 is the dominant HSPG in HK-2 cells, wild-type and Synd-1 knockdown (KD) HK-2 cells (Synd-1 KD) were compared in the following *in vitro* experiments:

#### Crotamine toxicity for cultured HK-2 PTEC cells

**Figure 5:**
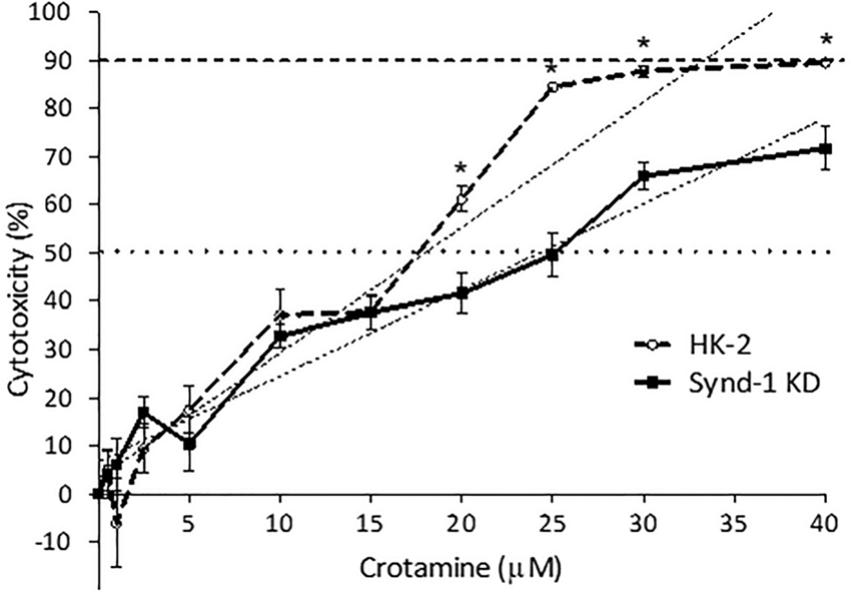
Cell viability assay for wild-type and Synd-1 deficient HK-2 cells (Synd-1 KD) treated with crotamine. Cytotoxicity of about 50% of wild type HK-2 cells was observed with ~18 µM of crotamine, while for Synd-1 KD cells, concentrations of ~25 µM of crotamine were required. Cytotoxicity for 90% of the wild-type HK-2 cells was observed with ~33 µM of crotamine, while a concentration higher than ~40 µM of crotamine would be required for Synd-1 KD cells. LC_50_: concentration of drug required to reach 50% of cytotoxicity (dotted line). LC_90_ concentration of drug required to determine 90% of cytotoxicity (dashed line).

Cell viability assay of wild-type and Synd-1 deficient PTEC HK-2 cells treated with several concentrations of crotamine (0–40 µM), for 72 h, demonstrated that crotamine LC_50_ for wild-type HK-2 cells was ~17.97 ± 0.73 µM, whilst for Synd-1 deficient HK-2 cells (Synd-1 KD) was ~24.53 ± 0.63 µM (*p* < 0.0001) (Fig. 5).

#### Crotamine interaction with and internalization by human PTEC HK-2 cells

**Figure 6:**
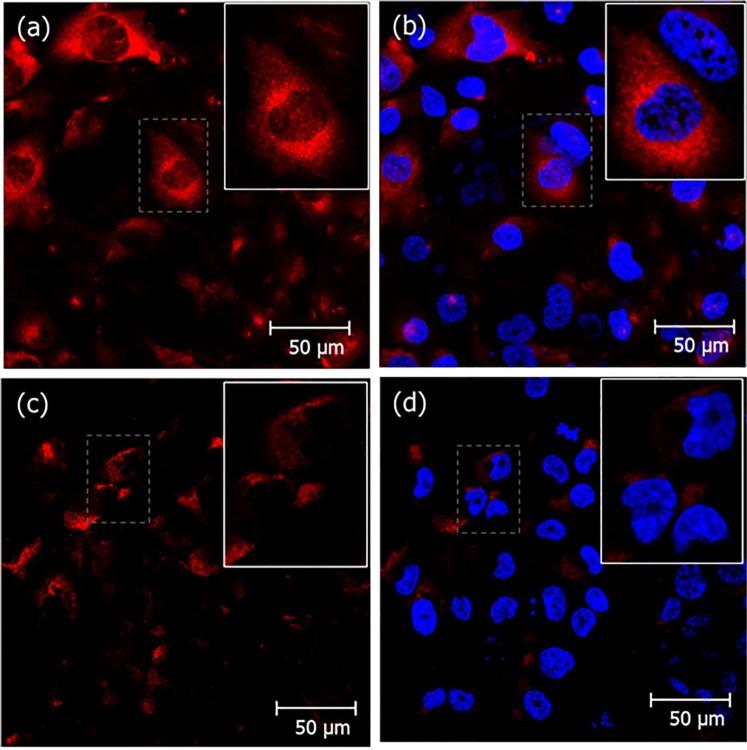
Reduced crotamine uptake in Synd-1 deficient HK-2 cells. Wild-type human PTEC HK-2 cells (**a**,**b**) and Synd-1 KD cells (**c**,**d**) treated with crotamine (5 μM) for 1 h, at 37 °C. Nuclei were stained with DAPI (in blue), and crotamine recognized by antibody after signal amplification with TSA-TRITC (in red). Crotamine (red) is visualized in the cytoplasm of wild-type human PTEC HK-2 cells, whilst in Synd-1 KD cells, significantly reduced fluorescence is noticed. Bar = 50 μm.

First we confirmed by FACS analysis (Supplementary Method S3) that the shRNA strategy reduced the expression of in PTEC HK-2 cells in about 80% (see Supplementary Fig. S5). The binding of Cy3-crotamine was also demonstrate to be ~3–4 times higher for wild-type human PTEC HK-2 cells compared with Synd-1 KD cells, which support the suggestion of a possible Synd-1 role in crotamine uptake by PTEC HK-2 cells (see Supplementary Fig. S5). In addition, confocal microscopy analysis allowed observing crotamine distributed in the cell cytosol, and accumulated in perinuclear area of HK-2 cells incubated with crotamine for 1 h (Fig. 6**A,B**), and this crotamine distribution pattern was similar to that in Synd-1 KD cells (Fig. 6**C,D**), but with a significant reduced intensity (~four fold less) (Supplementary Fig. S6).

#### Delivery of plasmid DNA into PTEC cells

**Figure 7:**
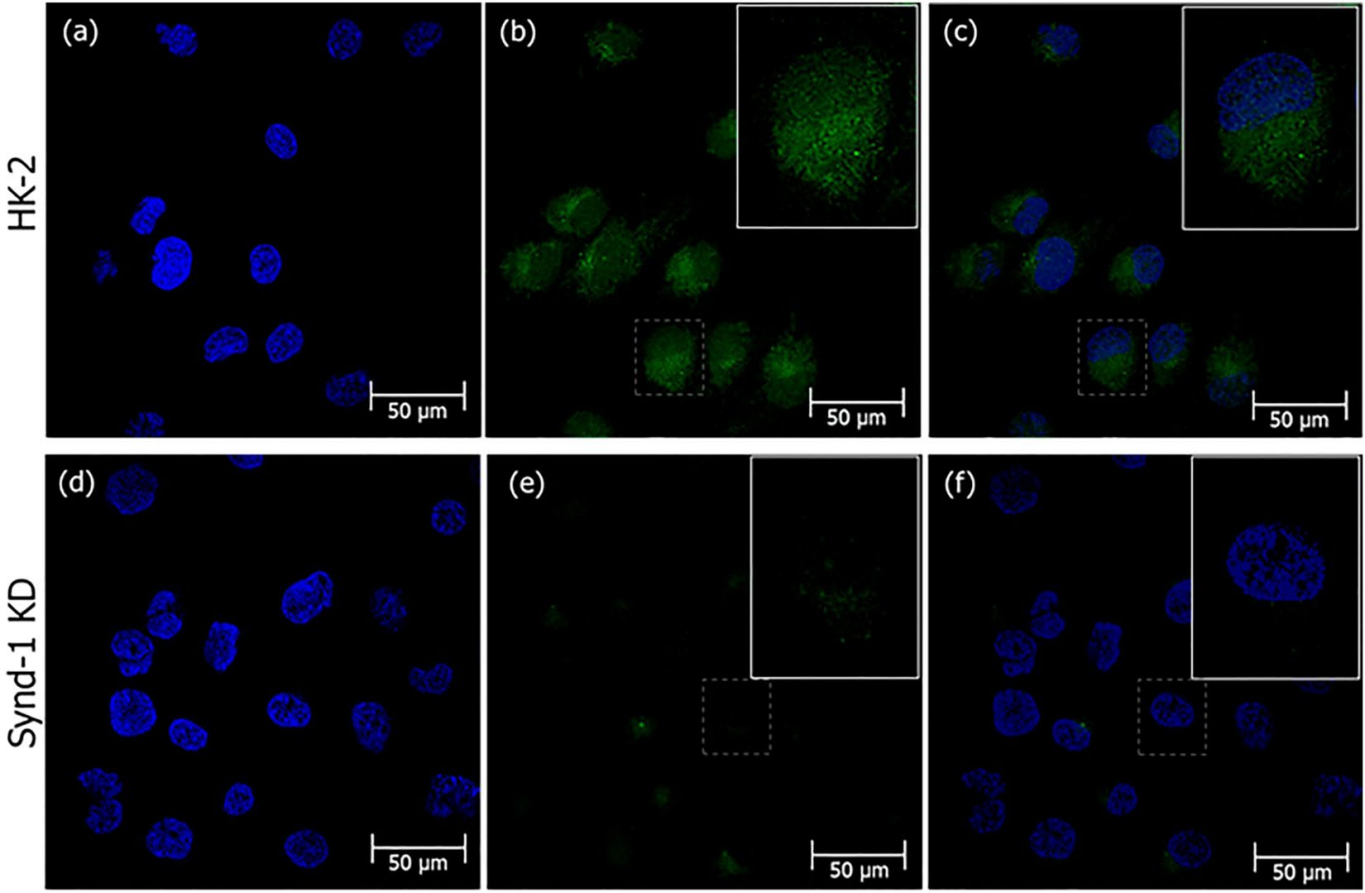
High transfection efficiency was observed in wild-type HK-2 cells for crotamine-mediated gene delivery and with reduced transfection in Synd-1 deficient HK-2 cells. Fluorescence confocal images of green fluorescent reporter gene expression in wild-type human PTEC cells (HK-2) (**a**–**c**) and Synd-1 KD cells (**d**–**f**) transfected by crotamine-mediated gene delivery. Nuclei stained with DAPI (in blue), GFP fluorescence (in green). Merged images (**c**,**f**). Bar = 50 μm.

To verify whether crotamine is able to translocate plasmid DNA into PTEC cells, a complex of crotamine and pEGFP-N1 plasmid vector was used to transfect wild-type HK-2 and Synd-1 KD cells. Crotamine-DNA complexes, formed by a peptide:DNA liquid charge ratio of 10:4, diluted in PBS solution was dropped onto semi-confluent cultured cells, and 24 h after, s strong green fluorescent signal, due to the plasmid DNA delivery and green fluorescent reporter protein (GFP) expression, was observed in wild-type HK-2 cells (Fig. 7), demonstrating the efficiency of transfection mediated by crotamine in this specific cell type. As expected, the green fluorescence signal was mainly localized in the cytoplasm of transfected cells, as similarly observed for Synd-1 KD HK-2 cells, but with significantly lower signal intensity (about 2.5 fold lower compared to wild-type cells) (Supplementary Fig. S6), reinforcing the contribution of the presence of HSPG Synd-1 for the internalization of crotamine-DNA complex.

## Discussion

In this communication we show that the *ip* injected CPP crotamine is partly reabsorbed by PTECs, and the full-length form of this polypeptide is partly excreted via urine. *In vitro* experiments with human PTEC cell line HK-2 suggest Synd-1 as one of the contributors for crotamine uptake into these cells. The possibility of oriented delivery of therapeutic compounds to PTECs would be of great interest for the treatment or intervention in different renal diseases with progressive tubulo-interstitial fibrosis such as diabetic kidney disease^36^.

It is worth to consider that after long-term daily administration of crotamine by *ip* injection for 21 days, crotamine main localization was noticed inside kidneys PTECs, but with no detectable noxious effect, as suggested by the general analysis of tissues (as liver and heart, among others) and cells morphologies by H&E staining. In addition, general biochemical biomarkers (namely creatinine kinase and uric acid) assessments suggested the healthy functioning of kidneys with excretion of the full-length intact crotamine in the urine of animals receiving high doses of this peptide acutely (single dose of 30 µg/animal, 1.5 h before urine collection), although long-term treated mice receiving low doses of this peptide (1 µg/animal/day) did not present detectable amount of crotamine in the urine collected daily along the treatment. On the other hand, single *ip* administration of fluorescently-labeled Cy3-crotamine (5 µg/animal), 2 h before euthanasia, allowed observing its presence in the brush borders area of PTECs (Fig. 3) and in the urine (Fig. 1 and Supplementary Figs S1 and S2), suggesting that crotamine is reabsorbed from pro-urine by PTECs. Unfortunately, the non-quantitative character of the MALDI-TOF MS^37^ did not allow us to quantify the precise amount of crotamine in the urine herein.

The universally expressed isoform of the syndecan, syndecan-4, is known to bind and mediate the transport of the most frequently utilized cationic CPPs, as penetratin, octaarginine and TAT^38^. Various experiments performed here indicate the participation of Synd-1 in the crotamine internalization into HK-2 PTECs. Firstly, compared with wild type HK-2 PTEC, crotamine binding is largely diminished in Synd-1 KD cells (Supplementary Fig. S5). Secondly, the LC_50_ concentration of crotamine is higher for the Synd-1 KD cells (Fig. 5). Thirdly, the crotamine cellular uptake and cytoplasmic/perinuclear staining is also reduced in the Synd-1 KD cells (Figs 6, 7).

The crucial roles of the proteoglycans for crotamine internalization was previously shown by us using Chinese hamster ovary (CHO) knockout cells for proteoglycans, namely CHO-745^18^. However, although we have showed earlier that Synd-1 is the dominant HSPG on PTECs both *in vivo* and *in vitro*^30^, it is important to consider that the knockdown strategy in the current report via shRNA strategy did not promote the complete suppression of Synd-1 expression^30^, and strong reduced expression (about 80%) but not a complete suppression of Synd-1 expression was observed (see Supplementary Fig. S5). At this point, one also needs to consider that potentially other proteoglycans with the ability to bind to crotamine^18^ might be present and eventually over-expressed as compensation in these human PTEC HK-2 cells, possibly ensuring the internalization of this peptide into PTEC cells. Although the suppression of Synd-1 expression did not completely suppress the internalization of crotamine or of crotamine-DNA complexes in Synd-1 KD, the internalization was significantly diminished in this knockdown (KD) cells, as demonstrated here. Also, lower toxicity for crotamine was also observed in the Synd-1 KD cells compared to HK-2 cell line (Fig. 5). It is worth to mention here that the LC_50_ for the wild-type human kidney cells was also significantly higher compared to those determined for tumoral cell lines, including murine melanoma B16F10 cells, for which a LC_50_ of about 1 µM was described^19,34,39^. Lower cytotoxicity of crotamine against non-tumoral compared to tumoral cells was also previously described by the group^34,39^, and the higher availability of proteoglycans on tumoral cells surface was suggested to explain this specificity of crotamine for highly proliferating cells^18^. Importantly, crotamine is therefore non-toxic for PTECs at working concentrations, which was always below 5 µM in the present study. Also, LC_50_ for cytotoxic effect against human HK-2 PTEC cells far exceeds the concentrations needed for gene transfection.

Although we did not show the *in vivo* delivery of reporter and/or therapeutic genes in the mice kidney PTECs, the absence of any noticeable damage or noxious effects on targeted cells and tissues using the free purified native crotamine, in addition to the successful delivery and expression of reporter gene in HK-2 cells mediated by crotamine showed in the present work, strongly stimulate us to continue this work, aiming the optimization of the conditions for the effective transfection of PTECs *in vivo*. We also envision therapeutic possibilities for non-viral crotamine-based PTEC-specific inhibition of Rho kinase in acute renal injury or peri-operatively, in renal transplantation. Beneficial effects of a lysozyme-conjugated Rho kinase inhibitor on acute renal allograft rejection were earlier shown^40^.

The specific targeting by crotamine can be used to reach specificities of PTECs such as IL-22R1 renal expression which is exclusive of this cell type, as this interleukin protects against renal I/R injury by activating STAT3 and AKT ameliorating I/R-induced renal inflammation and tubular cell injury^41^. Mitochondrial targeting ability of crotamine^32^ may also motivate the exploration of its potential application in mitochondrial dysfunctions in proximal tubule cells, aiming to protect, for instance, from nephrotoxicity associated with progressive tubulo-interstitial disease^42^.

Different therapeutic strategies targeting PTECs are described in literature. Protein- and peptide-based carrier systems, polymeric carrier systems, folate and antibody fragments are examples of PTECs targeting approaches^43^. A variety of therapies aims the drug delivery into the luminal side of the tubular cells, as on the apical membranes of these cells are found different internalizing receptors, which are able to internalize small molecules and macromolecules, such as proteins that are filtered into the urine, obtaining efficient drug uptake. Two well-known endocytic receptors are the megalin and cubilin, both expressed on the apical membrane of proximal tubular cells^43^. Molecules with strong affinity toward megalin are used as targeting delivery mediators such as polymyxin B, a polypeptide antibiotic^44^ and aminoglycoside antibiotics^45^, but these compounds show nephrotoxicity risk even in therapeutic doses.

During years, conjugate delivery systems were tested as renal-specific delivery alternatives to reduce drug nephrotoxicity such as 2-glucosamine conjugated with prednisolone^46^, carboxylated polyvinylpyrrolidone carrier^47^, conjugation with streptavidin^48^ or low-molecular-weight proteins such as lysozyme^49^, but these strategies still present renal toxicity and cardiovascular side effects. At this point, the use of crotamine as alternative drug carrier shows the advantage of showing no nephrotoxicity at therapeutic doses, even if needed for long-term treatments.

## Conclusion

Long-term (21 days) continuous (daily) administration of crotamine (1 µg/animal) by *ip* route in mice was generally non-toxic to the animals, as no significant change could be observed in weight of body or main organs, or bone size of treated animals. In addition, crotamine is also non-toxic for PTECs or kidneys of treated animals at the working concentrations, as no lesions were observed after histopathological analysis with two different staining and also kidney function biomarkers were not altered after daily treatment with crotamine. In addition, it is worth to mention that the cytotoxic concentrations far exceed the concentrations needed for gene transfection. Unprecedentedly, the accumulation of crotamine in kidney, more precisely, in PTECs was confirmed both for acute and long-term administration of crotamine, and we suggest Synd-1 as one of the contributors for crotamine internalization in PTECs, as it is the most abundant proteoglycan present in this cell type. Assays with Synd-1 deficient cells showed lower internalization of crotamine and also lower reporter gene transfection mediated by crotamine compared to wild-type HK-2 cells, reinforcing this hypothesis. Therefore, we propose here crotamine as a powerful tool for non-viral delivery and transport of therapeutic molecules aiming the modulation of aberrant gene and/or protein expression in PTECs, with no important noxious effects, to eventually slow down progressive tubulo-interstitial fibrosis.

## Materials and Methods

### Crotamine and other reagents

Crotamine was purified in the Ribeirão Preto Medical School, (FMRP), São Paulo University (USP), by Prof. Dr. Eduardo Oliveira from commercially available *Crotalus durissus terrificus* venom, basically employing the protocol previously described^20^. Rabbit anti-crotamine antibody was produced and purified by affinity chromatography by Dr. Eduardo Oliveira. Details about specificity are given in Supplementary Method. S4 and Supplementary Fig. S3. All other reagents, when not specified in the text, were of analytical grade and were mainly purchased from Sigma-Aldrich Inc. (St. Louis, MO, USA). The overall yield and purity of crotamine isolated from rattlesnake crude venom was determined by amino acid analysis after acid hydrolysis as previously described^20^. Purified native crotamine was labeled with the Cy3 fluorescent dye (Thermo Fisher Scientific Inc., Waltham, MA, USA), strictly following the instruction of the manufacturer, as described^20^. After labeling, the excess of fluorescent dye was removed by centrifugal 3 K filter unit device (Centricon, Amicon, Millipore Corp, Billerica, MA, USA). The labeling yield was calculated by absorbance measuring accordingly to manufacturer instructions, as described in details by Hayashi and collaborators^20^.

### Animal treatment and sample collection

Animals (male C57Bl/6 mice) were from the Experimental Animal Laboratory (INFAR) of the Federal University of São Paulo (UNIFESP/EPM, SP, Brazil). Mice were housed under controlled temperature (19 °C), 12 h dark and 12 h light cycles, and with free access to food and water, as recommended by the Guidelines for Ethical Conduct in the Care and Use of Animals, from American Psychological Association and the Guideline of the Committee on Care and Use of Laboratory Animal Resources of National Research Council from United States of America. This project was approved by Ethic Committee for Animal Use (CEUA) of UNIFESP/EPM (approval No. 6237220116), and the experiments with the animals were performed in accordance with FELASA guidelines.

Male C57Bl/6 mice (14 weeks old, 25–30 g) were divided in two experimental groups (N = 5 each): (1) control group, which received daily, 100 μL of saline per animal by intraperitoneal (*ip*) injection; (2) crotamine-treated experimental group which received during 21 days, the purified native crotamine (1 µg/animal/day by *ip* injection) freshly diluted in 100 μL of saline at the time of injection, following essentially the same protocol previously employed by us for the antitumor therapy^34,35^. Food and water intake were monitored per group of five mice and the data represent the mean value of 24 h consumption assessment. At the end of the treatment (*i.e*. after 21 days), for the analysis of crotamine localization in tissues, two animals of the treated group received a single shot fluorescently-labeled Cy3-crotamine (5 µg/animal in 100 μL of saline *ip* injected), while two control mice received 100 µL of saline by *ip* route, 2 h before the euthanasia. All animals were then weighed using a semi-analytical balance and after animal euthanasia, tissues were removed and weighed, before they were prepared for subsequent histological analysis.

Blood and urine samples were also collected for analysis at the end of 21 days treatment 24 h and 1.5 h, respectively, after last administration of crotamine. In order to detect crotamine in urine samples, two naive mice received a single *ip* injection of native purified crotamine or fluorescently-labeled Cy3-crotamine (30 µg/animal) while two control mice received 100 µL of saline by *ip* route, 1,5 h before the urine collection. The total volume of blood collected from each animal (~0.5 mL) was placed in microtubes containing heparin sodium 100 I.U./mL, and the sample was centrifuged for 10 min at 300 × g at room temperature for plasma fraction (supernatant) collection. The urine collected was centrifuged for 10 min, at 2000 × g, at 4 °C, to remove sediments. Aliquots of plasma and urine were stored in 0.2 mL microtubes at −80 °C until analysis.

### Biochemical markers analysis in blood and urine

The levels of creatinine in plasma and urine were measured by alkaline picrate method, as described by Jaffé^50^. Levels of uric acid in plasma and urine were measured by Uricase-PAP method^51^. For this purpose, specific kits, namely uric acid (Ácido Úrico Monoreagente – K139) and creatine (Creatinina Enzimática – K161), both from BIOCLIN (São Paulo, SP, Brazil), were used according to the manufacturer’s instructions.

### SDS-PAGE electrophoresis and Western blotting in urine

The presence of crotamine in the urine was determined by sodium dodecyl sulfate polyacrylamide gel electrophoresis (SDS/PAGE), Western blot analysis and mass spectrometry (MS) analysis (Supplementary Method. S1), which also confirmed the integrity of detected crotamine. For SDS-PAGE, 5 µL of urine samples of mice receiving vehicle (control), native crotamine (1 µg/animal/day) for 21 days, or acute administration of native crotamine (30 µg/animal). For the gel loading control, 100 ng of native purified crotamine were used. All samples were mixed with 2 × sample buffer (2% SDS, 10% glycerol, 0.1% bromophenol blue, 50 mM Tris pH 6.8), before denaturation by heating at 95 °C, and separation by electrophoresis in 15% SDS-PAGE, followed by Coomassie blue or silver staining. Another gel was prepared exactly in the same way, before it was transferred to nitrocellulose membrane (Hybond ECL; GE HealthCare, Little Chalfont, UK) for development by Western blot employing the anti-crotamine antibody (1:200) diluted in blocking buffer (3% bovine serum albumin), essentially as previously described^52^. The employed anti-rabbit IgG conjugated with alkaline phosphatase (Promega, Wiscosin, USA) secondary antibody was diluted 1:10,000 in TBST buffer (150 mM NaCl, 20 mM Tris-HCl pH 7.5 and 0.05% Tween-20), and a solution of 5-bromo-4-chloro-3-indolyl phosphate (BCIP) and nitro blue tetrazolium (NBT) in dilution buffer (0.10 M Tris–HCl pH 9.5, 0.02 M NaCl and 0.005 M MgCl_2_), strictly following the manufacturer’s protocols, was employed for the development of the immunorecognized protein bands. Imaging was captured by digital camera SM-G950x (Samsung, Seoul, South Korea) with the following specifications, ISO: 100, exposure time: 1/60 s, and focal length: 4.20 mm.

### Histopathological analysis of kidney

Kidneys were fixed in 4% paraformaldehyde before embedment in paraffin. Tissue sections (5–7 μm thick) were obtained from the paraffin-embedded blocks and they were mounted onto glass microscope slides after stretching at 40 °C. After deparaffinization, sections were stained with H&E and PAS for routine light microscopy examinations. Sections of kidneys from mice receiving Cy3-crotamine were deparaffinated and only incubated with DAPI followed by embedment with Citifluor^TM^ AF1 Mounting Medium (EMS Acquisition Corp., Pennsylvania, USA), before fluorescence confocal microscopy analysis.

### Immunohistochemistry

For the immunohistochemical analysis, tissue sections (3–4 μm thick) were prepared on a microtome from paraffin-embedded blocks, and they were mounted on superfrost glass slides, and dried overnight at 60 °C. Slides were deparaffinized with xylene/ethanol and rehydrated in PBS. Then, an antigen retrieval protocol was performed with Tris*-*HCl buffer pH 9.0, at 95 °C for 20 min. All further immunohistochemistry steps were carried out at room temperature (25 °C). Endogenous peroxidase was blocked with 0.03% H_2_O_2_ in phosphate buffer solution (PBS) for 30 min. To prevent non-specific binding of the antibodies to the tissues, the sections were incubated with 5% normal goat serum in PBS for 30 min. Rabbit anti-crotamine was used as primary antibody (diluted 1:2000 in 1% BSA*/*PBS), which was incubated on the tissue sections for 1 h. To control for specificity rabbit anti-crotamine in 1% BSA*/*PBS was blocked with 25 µg/mL of crotamine for 1 h prior to staining procedure. Goat anti-rabbit IgG conjugated with horseradish peroxidase (HRP; DAKO, Glostrup, Denmark) (1:100 in 1% BSA*/*PBS) was used as secondary antibody, which was incubated for 30 min, before the 10 min incubation in the dark with Tyramide Signal Amplification (TRITC-labeled tyramide solution, tetramethyl rhodamine system, Perkin Elmer, Waltham, MA, USA), employed to show the localization of the HRP-label by immunofluorescence. For nuclear staining, sections were incubated for 10 min in the dark with 300 nM DAPI (4′,6-diamidino-2-phenylindole), and embedment was done by Citifluor^TM^ AF1 Mounting Medium (EMS Acquisition Corp., Pennsylvania, USA) before fluorescence confocal microscopy analysis.

### Cell culture experiments

Human renal epithelial HK-2 cells were from ATCC^®^ (CRL-2190^TM^) and syndecan-1 deficient HK-2 cells were obtained by shRNA technology and selected based on zeocin resistance^30^. Cells were cultured in a mixture of DMEM and Ham’s F-12 medium, supplemented with 10 ng/mL human recombinant epidermal growth factor (EGF), 36 ng/mL hydrocortisone, 5 μg/mL bovine insulin, 5 μg/mL human transferrin, 5 ng/mL sodium selenite, 2 mM glutamax (Life Technologies, Carlsbad, CA, USA), 100 U/mL penicillin, and 100 μg/mL streptomycin.

### Cell viability assay

Cell viability after exposure to crotamine was examined using the MTT assay, in which metabolically active mitochondrial dehydrogenase activity converts the tetrazolium salt 3-[4,5-dimethylthiazol-2-yl]-2,5-diphenyltetrazolium bromide to insoluble purple formazan crystals at a rate proportional to cell viability. Cultured Synd-1 deficient and wild type HK-2 cells were plated in 96-wells microtiter plates, at a density of 2,500 cells/well, in 200 μL of culture medium (density was established by absorption curve and growth curve evaluation). After overnight incubation at 37 °C, the cells were incubated with crotamine (at concentrations ranging from 0 to 50 μM), in 200 μL of culture medium for 3 days, at 37 °C. At the end of the incubation, 20 μL of MTT solution (5 mg/mL in PBS) was added to each well. After 4 h, the plates were centrifuged at 900 rpm for 15 min, the culture medium was removed and 100 μL of DMSO 100% was added to each well and thoroughly mixed before the plate was read at 520 nm on a FlexStation3 microplate reader (Molecular Devices, Sunnyvale, CA, USA). Percentage of cytotoxicity was calculated using the following equation 100 × (1 − [optical density at 520 nm with crotamine]/[optical density at 520 nm without crotamine]). The assay was performed twice each of them in triplicate.

### Crotamine internalization assay

Analysis of crotamine internalization by PTEC HK-2 cells, cultured on glass cover slips, was performed by incubation of the cells with 5 μM of native non-labeled crotamine, at 37 °C for 1 h. After rinsing with PBS, the cells were fixed with 2% paraformaldehyde in PBS for 20 min, at room temperature. All further immunocytochemistry steps were carried out at room temperature (25 °C). Cover slips were mounted and evaluated for fluorescence signals by fluorescence microscopy.

### Crotamine transfection assay

Wild-type human HK-2 cells and Synd-1 deficient HK-2 cells (Synd-1 KD) were plated on 6 × 35 mm well plates using appropriate cell culture media. One day before transfection the cells were plated on glass slides at a density reaching about 50% confluence in 24 h. The crotamine-DNA complexes were prepared with plasmidial vector pEGFP-N1 (Clontech, Mountain View, CA, USA), containing a gene coding for GFP, essentially as previously described^18,20^. On the transfection day, the peptide-DNA complexes solution was added dropwisely to the cultured cells, and the cells with the peptide-DNA complex were incubated for 48 h. Cells were then fixed and stained with DAPI, before analysis in confocal microscopy.

### Confocal microscopy analysis

For immunohistochemical, immunocytochemical and transfection analysis, data acquisition was performed in Leica DM4000B fluorescence microscope (Leica Microsystems) or Leica TCS SP8 confocal microscope (Leica Microsystems), using 40× and 63× objectives, Leica TCS SP8 CARS – Coherent Anti-Stokes Raman Scattering (Leica Microsystems, Wetzlar, Germany) using 63× objective or IN Cell Analyzer 2000 (GE Healthcare, Little Chalfont, UK), using 2× objective. The parameters used were λ_EX_ 545 nm and λ_EM_ at 590–620 nm for Cy3-crotamine or TRITC, λ_EX_ 475–495 nm and λ_EM_ at 520–560 nm for GFP and λ_EX_ 405 nm/λ_EM_ 420–470 nm for DAPI fluorescence for microscopes or LED light source with TRITC filter for IN Cell Analyzer.

### Statistics

Data were analyzed using Student’s t-test comparing two groups (namely control and crotamine-treated groups). Results are presented as mean ± SEM with *p* < 0.05 considered as statistically significant.

## Supplementary information

Supplementary Dataset 1

## Data Availability

All data presented in this report are available for those who are interested in.
