# Supplementary material for: Long term safety of targeted internalization of cell penetrating peptide crotamine into renal proximal tubular epithelial cells in vivo
Source: Sci Rep. 2019 Mar 1;9:3312. doi: 10.1038/s41598-019-39842-7 (PMC6397221; doi:10.1038/s41598-019-39842-7)
Supplement: Supplementary file 1 — Supplementary Dataset 1 [file 41598_2019_39842_MOESM1_ESM.docx]

**Long term safety of targeted internalization of cell penetrating peptide crotamine into renal proximal tubular epithelial cells *in vivo***

Joana D’Arc Campeiro*^1^*, Wendy Dam^2^, Gabriela Monte*^1^*, Lucas Carvalho Porta*^1^*, Lilian Caroline Gonçalves de Oliveira*^3^*, Marcela Bego Nering*^1^*, Gustavo Monteiro Viana*^4^*, Fernando Cintra Carapeto*^5^*, Eduardo Brandt Oliveira*^6^*, Jacob van den Born*^2,*^*, Mirian A. F. Hayashi*^1,*^*

**Supplementary Information**

**Supplementary Methods**

**S1 Mass spectrometry analysis**

Mass spectrometry (MS) analysis were performed on a Microflex LT^TM^ (Bruker Daltonics, Bremen, Germany) instrument, and using the software FlexControl^TM^ version 3.4 (Bruker Daltonics). Briefly, 1 μL of 1:100 water diluted urine of mice treated with vehicle (control) and of mice treated with a single injection 30 µg of crotamine was spotted directly on stainless sample target plate and then air dried, before being overlaid with 1 μL of matrix solution (saturated solution of α-cyano-4-hydroxy cinnamic acid in organic solvent [50% acetonitrile and 2.5% trifluoroacetic acid (TFA)]), followed by instrument analysis. As positive control, 1 μL of purified crotamine (10 µg/mL) diluted 10 fold in water was spotted directly on stainless overlaid with 1 μL of matrix solution, before MS analysis.

**S2 SDS-PAGE electrophoresis and Western blotting of fluorescently-labeled Cy3-crotamine**

For SDS-PAGE, 5 µL of urine samples of mice treated with vehicle (control) or with acute injection of fluorescently-labeled Cy3-crotamine (30 µg/animal) and as control, 100 ng of fluorescently-labeled Cy3-crotamine were mixed with sample buffer (2% SDS, 10% glycerol, 0.1% bromophenol blue, 50 mM Tris pH 6.8), before denaturation by heating at 95°C, and electrophoresis in 15% SDS-PAGE followed by Cy3 fluorescence detection. Another gel prepared exactly in the same manner was transferred to nitrocellulose membrane (Hybond ECL; GE HealthCare, Little Chalfont, UK) before being developed by Western blot analysis employing the anti-crotamine antibody (dil 1:200), essentially as previously described.^51^ The secondary goat antibody anti-rabbit IgG conjugated with horseradish peroxidase (GE HealthCare) was diluted 1:10,000 in TBST buffer (150 mM NaCl, 20 mM Tris-HCl pH 7.5 and 0.05% Tween-20), and the development employed Amersham™ ECL™ Prime Western Blotting Detection Reagent (RPN2232; GE Healthcare, Little Chalfont, UK). Imaging was performed with ImageQuant LAS 4000 (GE HealthCare) using chemiluminescence method for peroxidase detection and green light filter (520 nm) for Cy3 fluorescence detection.

**S3 Flow cytometry assay**

For flow cytometry studies, wild-type and syndecan-1 deficient HK-2 cells were both plated in 12-wells cell culture plates. After overnight incubation at 37°C, cells were incubated with non-enzymatic cell dissociation solution 1X (C5789 - Sigma^®^), 600 µL/well, at 37ºC until cells were detached. Then, the cells were transferred to 4.5 mL tubes containing 2 mL cell medium, and after spin down by centrifugation at 300 ×g, for 5 min at 4ºC, the cells were washed 2 times with 2 mL of ice cold FACS buffer (1% BSA in PBS). Then, cells were incubated with fluorescently-labeled Cy3-crotamine (0 - 10 µM) on ice or with Alexa Fluor^®^ 647 mouse anti*-*human syndecan-1 (which is CD138; Bio-Rad/ AbD Serotec, California, USA) (dil. 1:100 and 1:200) for 1 h in FACS buffer, and after washed 2 times with 2 mL of ice cold FACS buffer. Thereafter, the cells were ressuspended in 300 µL of FACS buffer and kept on ice for FACS analysis in a FACSCalibur™ (Becton Dickinson, New Jersey, USA).

**S4 Enzyme-linked immunosorbent assay**

Nunc MaxiSorp ELISA plates (Nunc, Roskilde, Denmark) were coated overnight with 1 µg/mL of native crotamine in PBS. After blocking (PBS/5% bovine serum albumin; 2 h RT), wells were incubated for 2 h at RT with indicated concentrations of rabbit anti-crotamine or rabbit IgG in blocking buffer. Wells were washed (PBS/0.05%Tween-20) and incubated with goat anti-rabbit IgG conjugated with horseradish peroxidase (HRP; DAKO, Glostrup, Denmark) diluted 1:1000 in blocking buffer. Wells were extensively washed and substrate solution (3,3’,5,5’-tetramethylbenzidine) was incubated for 15 min at RT, after which the reaction was stopped using 10% H_2_SO_4_. Absorbance was read at 450 nm.

**Supplementary Figures**


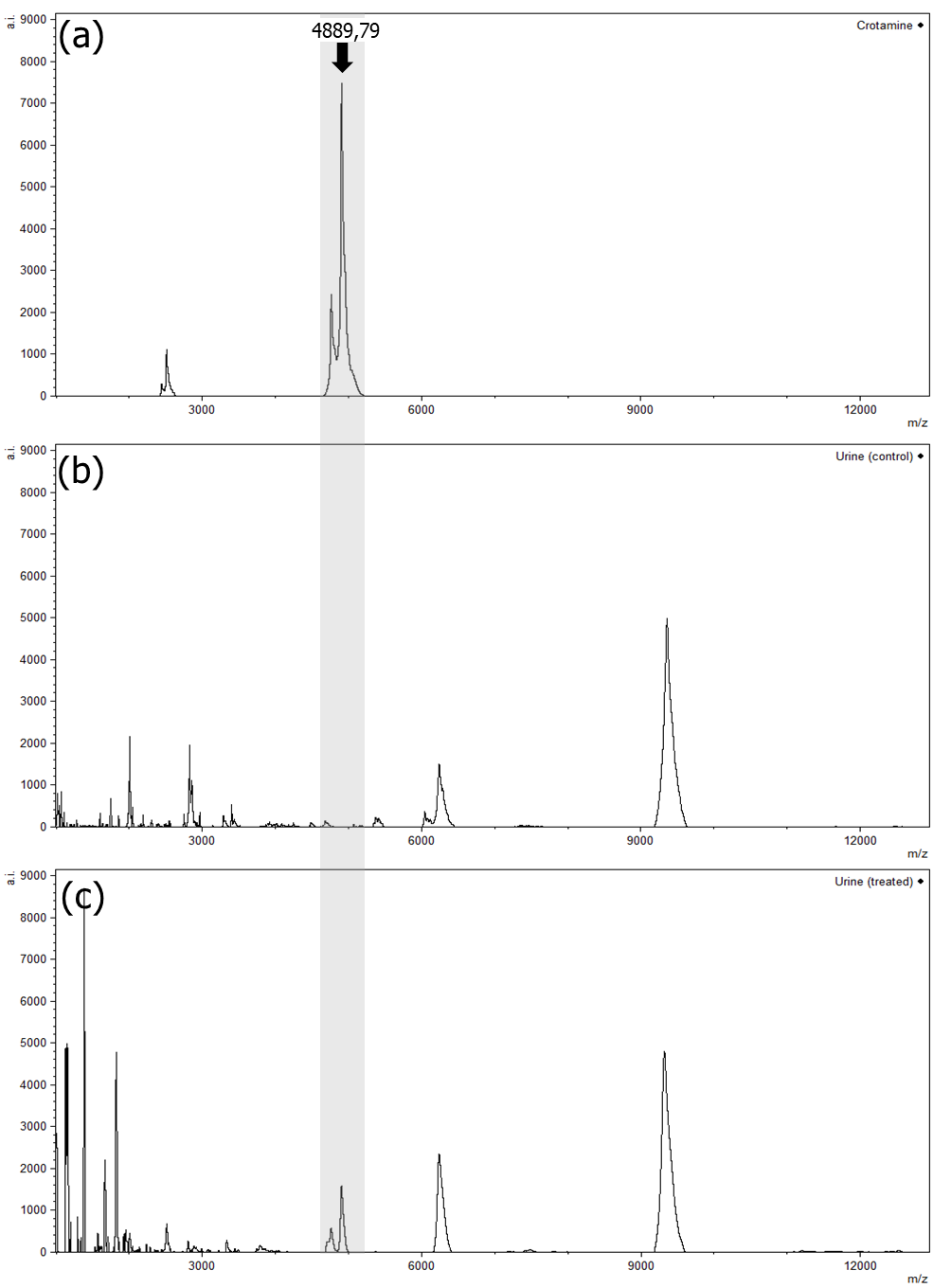


**Supplementary Figure S1. Detection of urinary crotamine by MALDI-TOF.** Mass spectrometry analysis of pure crotamine **(a)**, and urine of mouse receiving vehicle **(b)** or crotamine **(c)**. Mice received a single injection of crotamine (30 µg/animal) or vehicle by intraperitoneal (*ip*) route, and the urine samples were collected 1.5 h after. Arrow indicate the peak corresponding to crotamine molecular mass.


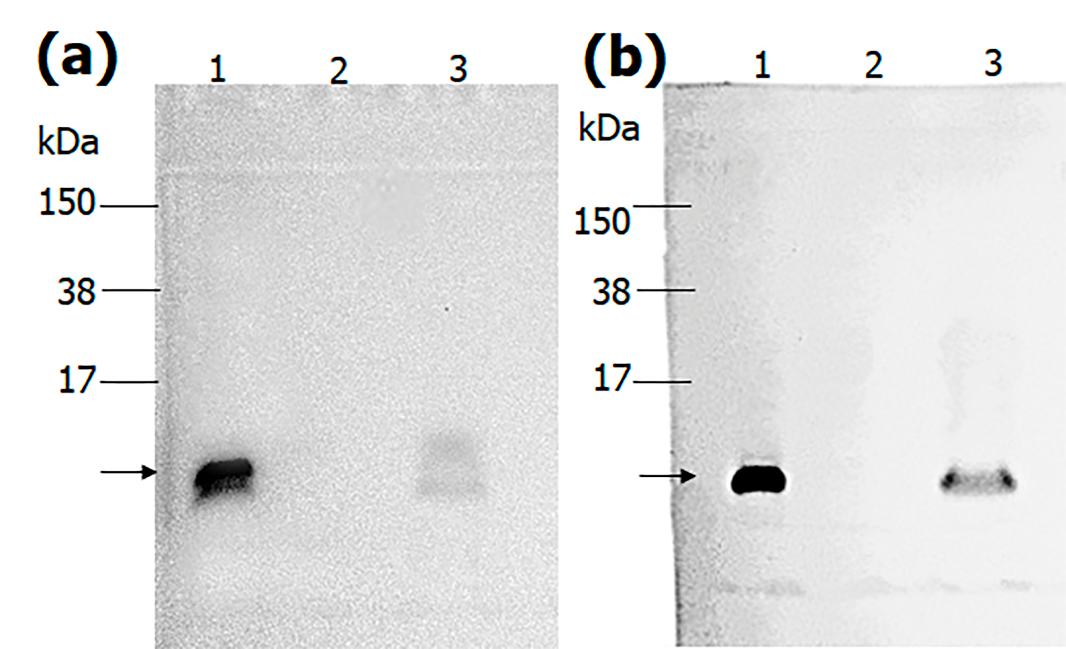


**Supplementary Figure S2. SDS-PAGE and Western blot analysis of urine samples.** Mice received a single injection of vehicle or Cy3-crotamine (30 µg/animal), by intraperitoneal (*ip*) route, 1.5 h before urine collection. **(a)** 15% SDS-PAGE showing Cy3 fluorescence emission, and **(b)** Western blot analysis with anti-crotamine antibody. Arrows indicate the protein band of about 5 kDa corresponding to the full-length crotamine. Lane 1: Cy3-crotamine (100 ng), lane 2: urine of a drug-naïve mouse receiving a single injection of vehicle (100 µL/animal), lane 3: urine of a drug-naïve mouse receiving a single injection of Cy3-crotamine (30 µg/animal). Urine samples were collected 1.5 h after the administration of vehicle or crotamine. For each lane, 5 µL of urine sample was loaded.

**
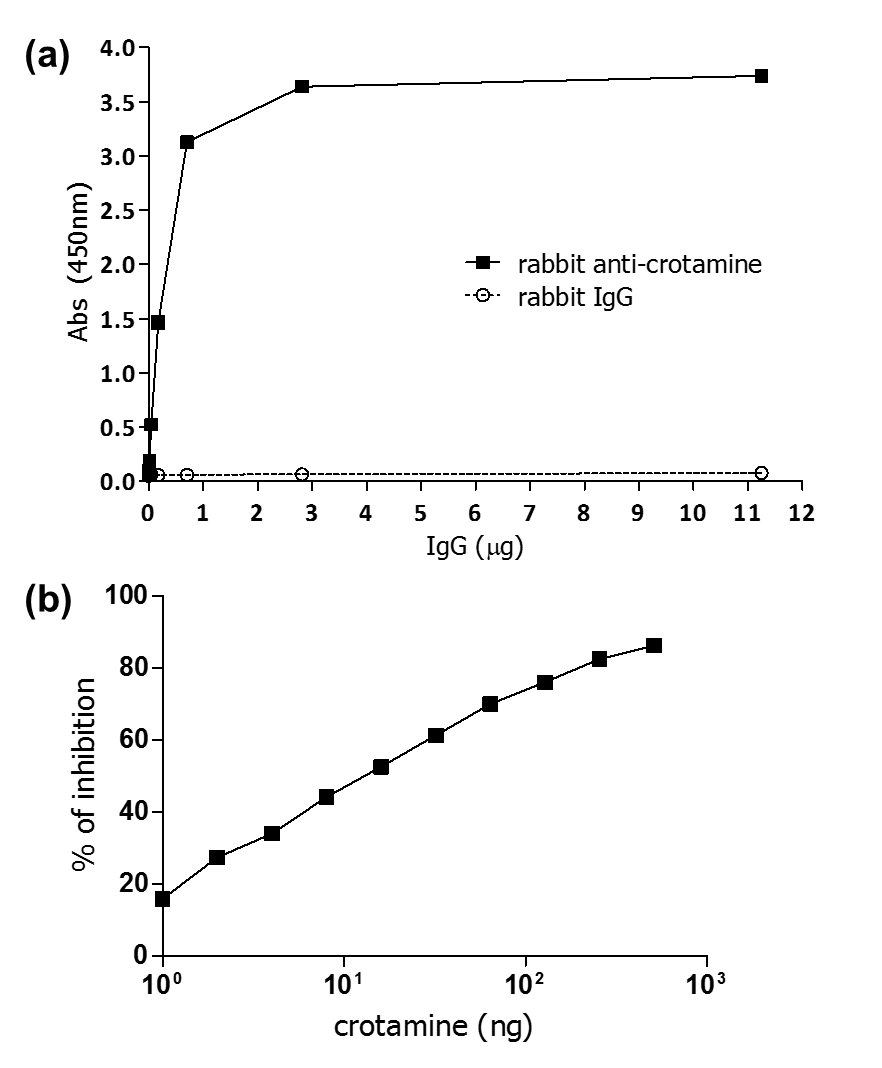
**

**Supplementary Figure S3. Enzyme-linked immunosorbent assay (ELISA) for evaluating the specificity of rabbit anti-crotamine**. (**a**) Rabbit anti-crotamine versus rabbit IgG on crotamine-coated wells. (**b**) Antibody inhibition with rabbit anti-crotamine incubated with different concentrations of crotamine as inhibitor.


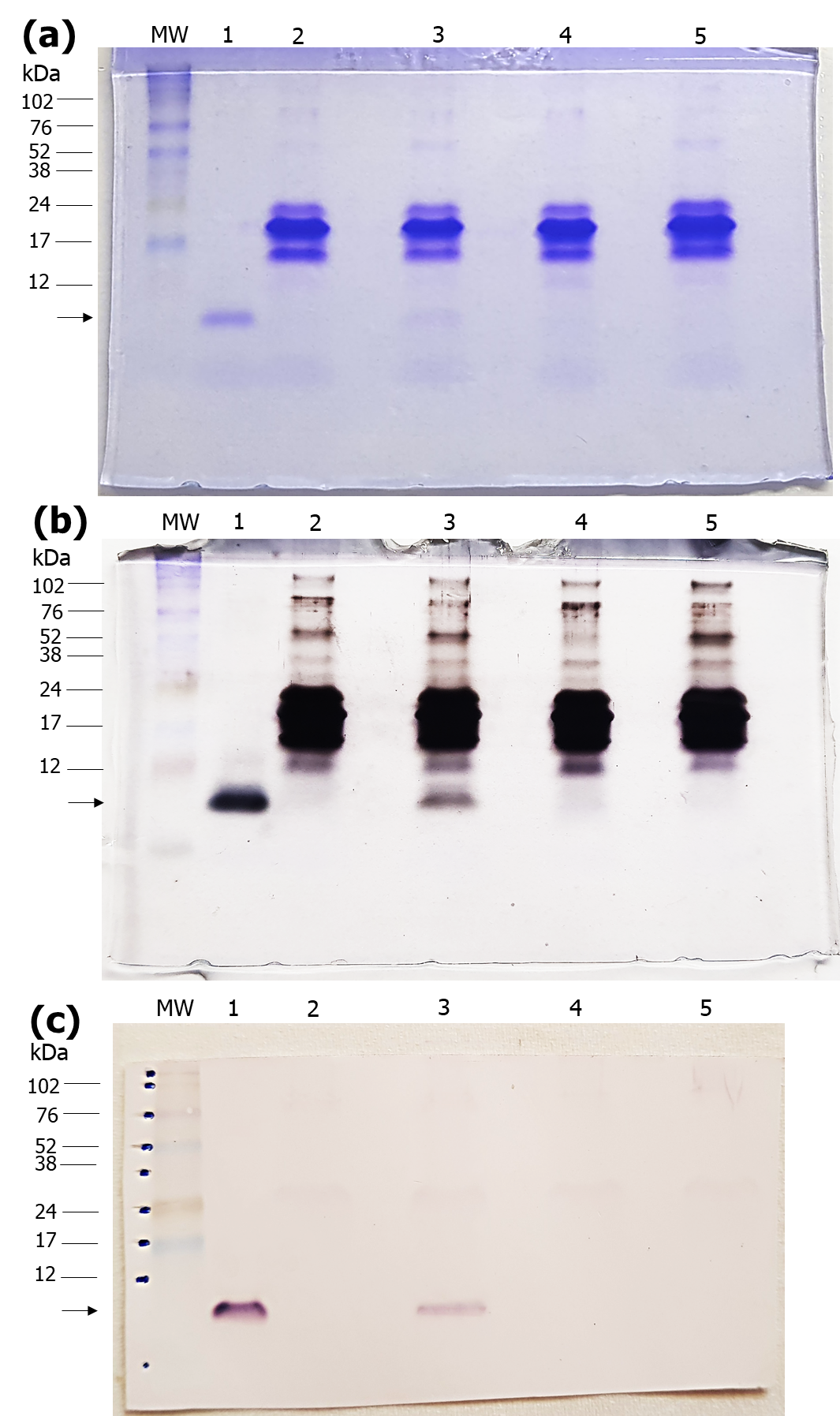


**Supplementary Figure S4. Raw images of Figure 1: SDS-PAGE and Western blot analysis of mice urine samples.** Urine of mice receiving vehicle saline or crotamine, by intraperitoneal (*ip*) route, were applied to a 15% SDS-PAGE and the gel was stained with **(a)** Coomassie blue or **(b)** silver, or the gel was transferred to nitrocellulose membrane for **(c)** Western blot analysis with anti-crotamine antibody followed by development with alkaline phoshatase and cromogenic substrates NBT/BCIP. The presence of a protein band of about 5 kDa, corresponding to the full-length crotamine, is indicated by the arrows. MW: molecular weight markers (kDa), lane 1: native crotamine (100 ng), lane 2: urine of drug-naïve mouse receiving a single injection of vehicle saline (100 µL/animal), lane 3: urine of drug-naïve mouse receiving a single injection of native crotamine (30 µg/100 µL/animal), lane 4: urine of negative control drug-naïve mice receiving vehicle daily (100 µL/animal) for 21 days, and lane 5: urine of mice treated daily with crotamine (1 μg/100 µL/animal) for 21 days. Urine samples were collected 1.5 h after the administration of vehicle or crotamine. In each lane, 5 µL of urine sample were loaded.

**Supplementary Figure S5. Syndecan-1 mediated crotamine binding to PTEC cell line HK-2.** Flow cytometry analysis of syndecan-1 (Synd-1) expression (**a** and **c**) and crotamine binding (**b** and **d**) for wild type (**a** and **b**) or Synd-1 knockdown (Synd-1 KD) (**c** and **d**) human PTECs. GeoMeanX reflects the MFI (Mean Fluorescence Intensity).

**
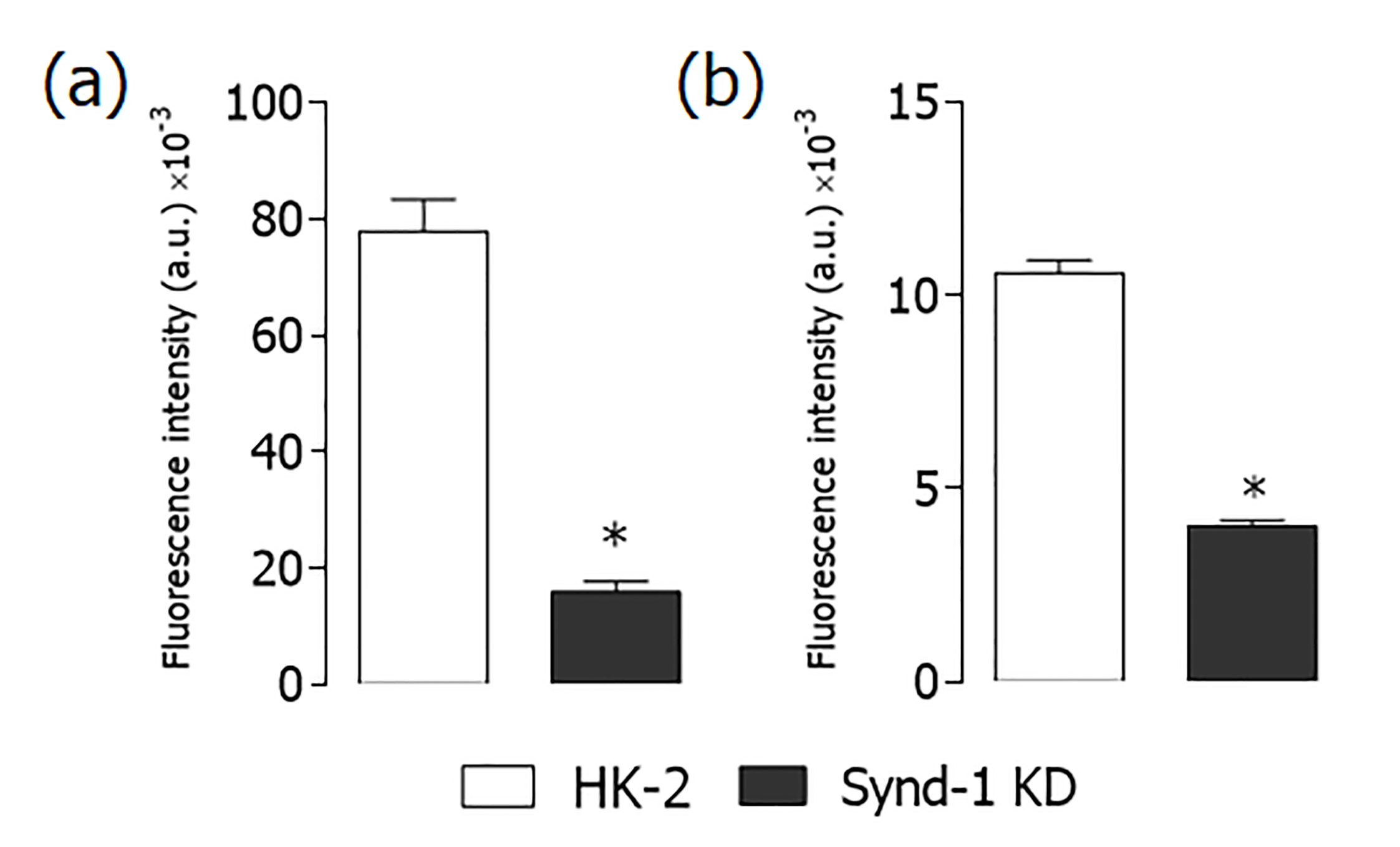
**

**Supplementary Figure S6. Quantification of relative fluorescence intensity**. Crotamine uptake in wild type and syndecan-1 knockdown (Synd-1 KD) HK-2 cells immunorecognized by antibody and followed by signal amplification with TSA-TRITC (a), and transfection of wild type and Synd-1 KD HK-2 cells with crotamine/pEGFP complex (b). The relative fluorescence intensity (a.u. = arbritrary unit) was determined by ImageJ software (N = 50 cells). **p* < 0.0001 for t-Student statistical test.
